# OSTEOSYNTHESIS OF A SCAPHOID NECK FRACTURE WITH A CANNULATED COMPRESSION SCREW: EVALUATION OF 52 PATIENTS

**DOI:** 10.1590/1413-785220233105e264116

**Published:** 2023-10-23

**Authors:** LUCAS BERNARDO CARVALHO DE ALMEIDA, VICTOR MARTINS MANFREDI, IGOR ARTHUR PARRON COSTA, FÁBIO SANO IMOTO, EIFFEL TSUIOSHI DOBASHI, THIAGO BERNARDO CARVALHO DE ALMEIDA, LUCIANO MILLER REIS RODRIGUES

**Affiliations:** 1 Rede D’Or, Hospital IFOR, São Bernardo do Campo, SP, Brazil; 2 Rede D’Or, Hospital IFOR, Residência Médica em Cirurgia da Mão, São Bernardo do Campo, SP, Brazil; 3 Rede D’Or, Hospital IFOR, Residência Médica em Ortopedia e Traumatologia, São Bernardo do Campo, SP, Brazil; 4 Universidade Federal de São Paulo, Escola Paulista de Medicina, Departamento de Ortopedia e Traumatologia, São Paulo, SP, Brazil; 5 Universidade Federal de São Paulo, Escola Paulista de Medicina, São Paulo, SP, Brazil

**Keywords:** Osteosynthesis, Wrist Fractures, Scaphoid Bone, Osteossíntese, Fraturas do Punho, Osso Escafoide

## Abstract

Objective: To evaluate the effectiveness of the dorsal fixation technique with a cannulated compression screw (CCS) for transverse scaphoid neck fractures. Methods: A case series study was carried out with patients treated with a CSS between April 2014 and May 2021. The main outcome was the healing of the fracture, verified by radiographic evaluation that used images of the wrist in anteroposterior, lateral, radial deviation, ulnar deviation and oblique views, obtained in the postoperative period. Results: Fifty-two patients aged between 15 and 65 years were analyzed, of which 43 (83%) were male. Of the 52 patients, 19 (36.53%) had a right-hand injury and 33 (63.46%) had a left-hand injury. Results were excellent in 47 patients (90.38%); good in 4 patients (7.69%), with reduced mobility compared to contralateral and poor in 1 patient (1.92%), with failure of consolidation and breakage of the synthesis material. In 51 cases (99%) there was bone consolidation at the end of six months. Conclusion: Osteosynthesis with a cannulated compression screw is a safe, effective and promising method for the treatment of scaphoid neck fractures. *Level of Evidence IV, Case Series.*

## RESUMO

Objetivo: Avaliar a eficácia da técnica de fixação dorsal com parafuso canulado de compressão (CCS) para fraturas transversas do colo do escafoide. Métodos: Foi realizado um estudo de série de casos com pacientes tratados com CSS entre abril de 2014 e maio de 2021. O desfecho principal foi a consolidação da fratura, verificada por meio da avaliação radiográfica das imagens do punho em anteroposterior, perfil, desvio radial, desvio ulnar e oblíquo obtidas no pós-operatório. Resultados: Foram analisados 52 pacientes com idade entre 15 e 65 anos, sendo 43 (83%) do sexo masculino. Dos 52 pacientes, 19 (36,53%) tinham lesão na mão direita e 33(63,46%) na mão esquerda. Os resultados foram excelentes em 47 dos pacientes (90,38%); bons em quatro (7,69%), com mobilidade reduzida comparada ao membro contralateral; e ruim em um (1,92%), com falha da consolidação e quebra do material de síntese. Em 51 casos (99%) houve consolidação óssea ao final de seis meses. Conclusão: A osteossíntese com parafuso canulado de compressão é um método seguro, eficaz e promissor para o tratamento das fraturas no colo do escafoide. *Nível de Evidência IV, Série de Casos.*

## INTRODUCTION

Scaphoid fractures are the most common carpal fractures and tend to occur in younger, more active individuals. The consolidation of fractures may be challenging due to their particularities and to the vascularization of the scaphoid.^1^ ^), (^ ^2^ Scaphoid fractures account for 2% to 7% of all fractures and for 60-70% of carpal injuries.^1^ ^), (^ ^3^

The most common injury mechanism for scaphoid fractures is falling with an outstretched hand.^2^ Delays in diagnosis or inadequate treatment can lead to nonunion, deformity and instability, which develop over five years. If left untreated, scaphoid nonunion can develop into carpal collapse and degenerative arthritis, leading to significant deficiency.^1^ ^), (^ ^2^ As these lesions usually occur in young and active patients, the associated morbidity and cost implications of the disability are significant.^1^ ^), (^ ^2^

Successful scaphoid fracture consolidation is not universal and depends on the type of fracture and use of appropriate surgical techniques.^2^ The role of the scaphoid within the structure and mechanics of the carpus, including directing and rotating the longitudinal axis of the scaphoid, is an important kinematic component to consider in scaphoid fracture repair.^3^ Due to the limited vascular supply, it is important to obtain adequate reduction and cure to avoid complications, including avascular necrosis.^3^

Percutaneous internal fixation of scaphoid fractures allows for a more predictable union, with less morbidity than a treatment with plaster or an open internal fixation. The surgical technique that uses a headless cannulated compression screw, implanted by means of a dorsal percutaneous approach with the aid of fluoroscopy and arthroscopy, is indicated for the correction of acute fractures and presents low complication rates.^4^ ^), (^ ^5^ Thus, the present study aimed to analyze the clinical results of 52 cases that used the cannulated compression screw (SCC) in the dorsal fixation technique for scaphoid neck transverse fractures.

## METHODS

This is a retrospective case series study conducted in a referral hand surgery hospital in the state of São Paulo. The investigation of the cases occurred through the analysis of medical records of patients submitted to osteosynthesis of transverse scaphoid neck fractures, with cannulated compression screw (CCS), by dorsal approach, in the period from April 1, 2014 to May 31, 2021.

This study was approved by the Research Ethics Committee of the São Luiz Hospital and Maternity, São Paulo, SP and received the opinion number 5,277,168 and CAAE: 564661322.0000.0087.

### Inclusion and exclusion criteria

Inclusion criteria were patients of both sexes, with a scaphoid neck fracture classified as B2 by the Herbert and Fischer scale for acute, unstable fractures (< 6 weeks), complete of the neck,^6^ ^)^ patients who underwent the scaphoid osteosynthesis by means of the CCS screw, from April 1, 2014 to May 31, 2021; clinical and radiographic follow-up of at least 6 months after the operation. Patients submitted to any other therapy modality of therapy, data from incomplete medical records and information on any other type of fracture other than the scaphoid were excluded.

### Surgical technique of osteosynthesis in the scaphoid neck fracture

All patients were placed in the supine position and submitted to brachial plexus block as anesthesia. The fractures were reduced by making a single horizontal incision on the dorsum of the wrist, distal about 1 cm from Lister’s tubercle and approximately 2 cm long. Cautious dissection was performed until the identification of the 3 extensor compartment, dissected and removed for the dorsal opening of the capsule.^7^ With the preparation of the surgical site, the identification of the articular surface of the scaphoid proximal pole, the fracture was reduced with the aid of radioscopy and osteosynthesis was made by the passage of 1 guide wire through the long axis of the scaphoid, perpendicular to the fracture trace, allowing for the passage of 1 self-perforating cannulated CCS screw.^8^ The capsule and incision were sutured after being cleaned with 0.9% saline, tourniquet loosening and a hemostatic control process, with dressing and placement of a plaster splint, used in the postoperative period for pain control. Patients were discharged 8 hours after the end of the intervention. The plaster splint and the stitches were removed in 2 weeks, and patients then started rehabilitation with the physiotherapy service that followed the established protocol. After 6 weeks, patients were submitted to an evaluation of the degree of satisfaction of the treatment, according to the criteria proposed by Crawford.^9^

### Analyzed outcomes

The main outcome observed was the consolidation of the fracture, analyzed through radiographic evaluation using anteroposterior, profile, radial and ulnar deviations and oblique wrist images obtained during the postoperative follow-up, performed in a period of 1 week, 2 weeks, 4, 6, 8, 12, weeks and 6 months. Bone consolidation criteria, for fractures analyzed through radiography, included ones that did not present signs of pseudarthrosis, that is, sclerosis at the extremities of the fracture, presence of a hiatus, absent or hypertrophic bone callus and persistence or enlargement of the fracture trait, after a minimum follow-up period of 6 months, were considered. In addition, the report of the presence of pain was evaluated in all patients.

### Statistical analysis

A database was built using the Microsoft Excel program. For the analyses, the statistical package Stata version 13.0 (Stata Corp LP, College Station, TX, USA) was used. All values were presented in percentages and in a pie chart.

## RESULTS

**Figure 1:**
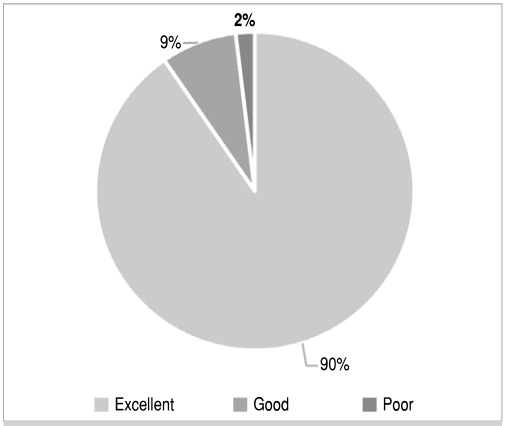
Scaphoid fracture consolidation distribution chart.

In total, 52 patients were screened in the study. Their ages ranged from 15 to 65 years and they underwent surgical treatment for scaphoid fractures. Nine (17%) patients were female and 43 (83%) were male. Of the 52 patients, 19 (36.53%) were injured in the right-hand while 33 (63.46%) were injured in the left-hand. The results obtained were excellent for 47 of the patients (90.38%), good for 4 patients (7.69%), who had reduced mobility compared to contralateral, and poor in 1 patient (1.92%), with consolidation failure and breakage of the synthesis material (Figure 1).

**Figure 2:**
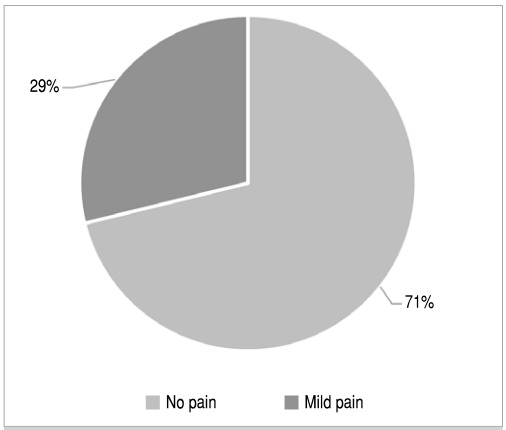
Distribution graph of pain prevalence after six months of surgery.

At the end of six months, 37 patients (71.15%) did not report any type of pain and 15 patients (28.85%) had mild pain (Figure 2).

## DISCUSSION

The scaphoid is the largest of the eight carpal bones and anatomically contains the proximal and distal poles with a waist (its mid-portion). The blood supply of the scaphoid is carried out predominantly by the radial artery branches and secondarily by the superficial palmar arch. Because of the retrograde nature of their blood supply, fractures in the scaphoid waist leave the proximal pole at high risk of avascular necrosis.^1^

The use of the surgical technique in which the cannulated compression screw is implanted through a dorsal percutaneous approach is indicated for the correction of acute fractures and delayed unions that are not associated with avascular necrosis or collapse.^4^ In this sense, the present study analyzed the clinical results of 52 patients who used the dorsal fixation with cannulated screw (CSS) technique. The results six months after surgical treatment indicated that this technique is a good option for fractures in the scaphoid neck.

The present study also indicated excellent results regarding fracture consolidation after six months of surgery, with no pseudarthrosis reports and good functional results. Folberg et al.,^8^ 2004, in a case series of 16 fractures of the middle third of the scaphoid, used percutaneous fixation with a cannulated Herbert screw as surgical treatment. The authors concluded that the percutaneous fixation technique in fractures of the middle third of the scaphoid proved to be an effective alternative, with low morbidity in the treatment of this lesion and no reports of complications throughout and after operations.^8^

In another study, with a retrospective review of medical records of 24 patients undergoing a surgery that involved fixation of non-displaced fractures (< 1 mm) of the scaphoid waist with a dorsal percutaneous cannulated screw, the overall rate of complications was 29%. There was one case of proximal scaphoid pole fracture after the operation. Minor complications included intraoperative equipment breakage - one case involving a screw and another involving a guide wire.^5^

In the percutaneous scaphoid fixation by dorsal route, the use of a limited incision is recommended when fixing the scaphoid internally by the dorsal approach, since there are anatomical structures at risk of injury.^10^ Authors indicate that percutaneous fixation is a valuable treatment method for scaphoid fractures, as it allows early wrist movement and high patient satisfaction.^10^ ^), (^ ^11^

The main limitation of the study is its design, a retrospective case report. We point out the need for further studies, including randomized clinical trials with the presence of a control group, to confirm our findings regarding scaphoid neck fracture osteosynthesis with cannulated screws.

## CONCLUSION

There was fracture consolidation in 99% of the cases after follow-up and good functional results. Scaphoid neck fracture osteosynthesis with a cannulated compression screw, by the dorsal route, is a safe, effective and promising method for the treatment of scaphoid neck fractures.
